# Editorial: Mechanisms of Prokaryotic Predation

**DOI:** 10.3389/fmicb.2020.02071

**Published:** 2020-09-08

**Authors:** David E. Whitworth, Edouard Jurkevitch, Juana Pérez, Gregor Fuhrmann, Susan F. Koval

**Affiliations:** ^1^Institute of Biological, Environmental and Rural Sciences, Aberystwyth University, Aberystwyth, United Kingdom; ^2^Department of Plant Pathology and Microbiology, Hebrew University of Jerusalem, Jerusalem, Israel; ^3^Department of Microbiology, Faculty of Science, University of Granada, Granada, Spain; ^4^Helmholtz-Institute for Pharmaceutical Research Saarland, Saarbrücken, Germany; ^5^Department of Microbiology and Immunology, University of Western Ontario, London, ON, Canada

**Keywords:** BALO, myxobacteria, prey, microbial ecology, *Bdellovibrio*

As organisms whose life-cycles are intrinsically antimicrobial, prokaryotic predators can profoundly affect the composition of microbiomes, including those underpinning human health, disease, agriculture, environmental, and industrial processes. Our understanding of prokaryotic predators has become increasingly mechanistic as technical advances have allowed us to probe whole cells, populations and ecosystems, identifying the genes and gene products responsible for predatory activity.

However, there is still much to learn about the molecular mechanisms of predation, the relevance of those mechanisms to predator biology/ecology and the potential for their exploitation to benefit humanity. The purpose of this Research Topic was therefore to gather together research and theories investigating the mechanisms of predation by prokaryotes.

The Research Topic has drawn together eight submissions, which together focus on the two best-studied prokaryotic predators, myxobacteria and BALOs (*Bdellovibrio* and like organisms).

In BALOs, motile “attack phase” cells penetrate the outer membrane of Gram-negative bacterial prey, forming a bdelloplast. The BALO cell elongates and replicates its DNA within the bdelloplast as it feeds upon the contents of the prey cell. However, cell division does not occur until prey nutrients have been depleted. When cell division does happen, it is by synchronous division of the filament into multiple progeny cells, with the number of progeny cells produced depending on the amount of nutrients within the original prey cell. Two papers in the Research Topic deal with this fascinating and relatively poorly understood aspect of BALO predation. In a Perspectives article, Laloux reviews what is known about predatory cell division, and suggests experiments and methods which have the potential to shed light on this strange and poorly understood phenomenon. In particular, studies are encouraged into cell polarity and the spatio-temporal organization of the proteins controlling non-binary cell fission. In the Original Research by Milner et al., the role of the cell division protein DivIVA was investigated in *Bdellovibrio bacteriovorus*. DivIVA was found to be expressed during filamentous cell division, and was shown to interact with proteins that potentially indicate nutritional status and prey nutrient levels. A protein encoded in the same operon as DivIVA was also shown to interact with the *B. bacteriovorus* ParA homolog, which regulates predatory cell division, priming future studies into partitioning and cell division within the bdelloplast.

Unlike BALOs, myxobacteria are epibiotic predators, secreting antimicrobial metabolites and proteins into the extracellular environment where they kill and digest prey cells from outside. Three pieces of Original Research in the Research Topic investigate molecular mediators of predatory activity in myxobacteria. Sutton et al. undertook a genome-wide association study to identify genes whose presence correlated with predatory activity. Among the genes they identified was that encoding formaldehyde dismutase (Fdm), which detoxifies the antimicrobial compound formaldehyde. Addition of Fdm increased the predatory activity of myxobacterial strains against formaldehyde-secreting *Pseudomonas aeruginosa*, suggesting that formaldehyde secretion is a predation-resistance trait of some prey organisms. The Original Research by Contreras-Moreno et al. shows that copper accumulates in the extracellular space between predatory *Myxococcus xanthus* and prey *Sinorhizobium meliloti* cells. The predator expresses copper-resistance genes, while the prey cells produce melanin, which can protect against oxidative stress. By disrupting genes required for copper-induced melanin production, it was shown that if *S. meliloti* is unable to produce melanin, it becomes more susceptible to predation by *M. xanthus*, suggesting melanin is another mediator of prey cell predation-resistance.

While nitrogen-fixing *S. meliloti* forms symbioses with legumes, promoting plant growth, myxobacteria are also generally considered to be plant growth promoting, by preying upon plant pathogens. Adaikpoh et al. investigated the response of myxobacterial predators to the plant phytohormone methyljasmonate. In their Original Research article, they showed that *Archangium* sp. strain Cb G35 responds systemically to methyljasmonate exposure with pervasive changes throughout its transcriptome and metabolome. The phytohormone caused an increase in swarm expansion and lytic enzyme production, which suggest that the plant uses methyljasmonate to recruit a subset of predatory myxobacteria to the plant.

It is clear from the Original Research articles described above that prokaryotic predator-prey interactions have the innate potential to impact substantially on plant growth. Understanding the mechanisms at play will allow us to rationally exploit prokaryotic predators as biological control agents, and in other applications. Such potential applications are the focus of the Review article by Bratanis et al. who highlight a variety of ways in which BALO biology and gene products could be used in diverse industrial and medical arenas. Given particular attention are the topics of secreted enzymes (especially proteases), biological control, biofilm formation/degradation and bioplastic production. The authors also discuss the potential for genetic engineering of predatory strains and the potential limitations of BALO applications. Potential future applications are also highlighted in the Mini-Review of myxobacterial predation by Thiery and Kaimer. Important aspects of myxobacterial predation considered by the authors include the mechanisms of motility, how prey cells are lysed, and the delivery of predation factors to prey cells. Myxobacteria are well-known for their population-wide multicellular behaviors, particularly fruiting body formation in response to starvation. Predation by myxobacteria can also be considered as a multicellular behavior, and the relationships between predation and fruiting body formation are discussed in the Mini-Review.

The ecological importance of prokaryotic predators is the topic tackled in the Original Research by Ezzedine et al. who investigated the distribution of BALOs in Lake Geneva. Using a metagenomic approach to assess microbial diversity, a seasonal and vertical distribution of BALOs was observed, with different taxa dominating at different depths and at different seasons. The abundance of BALO taxa was found to correlate positively with a number of non-BALO taxa including myxobacteria—potentially reflecting their taxonomic closeness, and/or their shared predatory lifestyle.

In conclusion, new insights gained by studies such as those included in this Research Topic on Mechanisms of Prokaryotic Predation are likely to be crucial in allowing us to rationally perturb microbial community composition, and deliver predation-inspired improvements in health, food security, industry, and the environment.

## Author Contributions

All authors listed have made a substantial, direct and intellectual contribution to the work, and approved it for publication.

## Conflict of Interest

The authors declare that the research was conducted in the absence of any commercial or financial relationships that could be construed as a potential conflict of interest.

